# Liver abscess in the caudate lobe caused by *Klebsiella pneumoniae*: a rare case report and literature review

**DOI:** 10.1186/s12879-024-09569-6

**Published:** 2024-07-19

**Authors:** Lingxia Cheng, Lei Li, Yongzao Liu, Wei Cheng, Guan Wang, Ping Xu

**Affiliations:** 1 Emergency Department, Zigong Fourth People’s Hospital, Zigong, China; 2 West China Hospital, Sichuan University/West China School of Nursing, Sichuan University, Chengdu, China

**Keywords:** Liver abscess, Caudate liver lobe, Sepsis, Invasive liver abscess syndrome

## Abstract

**Background:**

*K.* pneumoniae liver abscess (KPLA) mostly involves the right lobe. We present a case of *K. pneumoniae* caudate liver abscess with invasive liver abscess syndrome (ILAS) was rarely identified.

**Case presentation:**

A 53-year-old man with elevated glycated hemoglobin with chills, rigors and a fever of five days. The patient presented with tachycardia and fever. Physical examination revealed tenderness over the right abdomen was elicited. In particular, the inflammatory markers were markedly elevated, and computerized tomography (CT) showed pulmonary abscess, pulmonary embolism and caudate liver abscess. The patient’s sequential organ failure assessment (SOFA) score was 10 points. *Klebsiella pneumoniae* was isolated from sputum, urine and blood. With the suspicion of liver abscesses, ILAS and sepsis. The patient was successfully treated with antibiotics. He returned to close to his premorbid function.

**Conclusion:**

*K. pneumoniae* caudate liver abscess was rare. This is the first detailed report of *K. pneumoniae* caudate liver abscess with invasive liver abscess syndrome. Patients with cryptogenic *K. pneumoniae* liver abscess are advised to undergo an examination of intestinal barrier function. The study indicates that in patients with *K. pneumoniae* liver abscess, a caudate liver abscess size of ≤ 9.86 cm² may be characteristic of those suitable for conservative treatment of invasive liver abscess syndrome.

**Supplementary Information:**

The online version contains supplementary material available at 10.1186/s12879-024-09569-6.

## Background

Pyogenic liver abscess (PLA) is caused by various bacteria (e.g., *K. pneumonia, E. coli, Streptococcus*, etc.) [1], and a mortality rate of 2.8–31.8% in patients with liver abscesses [2, 3].

It has been determined that *K. pneumoniae* is the most common PLA pathogen in Asia [4], the range for the rate of metastatic infection is 3.5–20% [5]. Patients with KPLA had an 8 − 24% chance of developing ILAS. ILAS is deceiving because it commonly manifests as a metastatic infection with sites of infection including, but not limited to, the liver, eye, central nervous system, lungs, musculoskeletal system, and urinary tract [6]. ILAS involving the lung manifested as pulmonary infection, septic pulmonary embolism or lung abscess. The delayed diagnosis and treatment of KPLA patients with initially stable hemodynamics can result in rapid progression to sepsis [7]. PLA is associated with numerous risk factors, including diabetes, biliary tract disease and history of cancer [8]. The overall mortality in ILAS patients was significantly higher than those of the non-metastatic [9]. KPLA were more likely to appear as a single abscess cavity to involve single lobe and the right lobe was affected more frequently [7]. We hereby describe a rare case of *K. pneumoniae* caudate liver abscess with septic pulmonary embolism. We used initiation of continuous renal replacement therapy (CRRT) and antibiotics sequential therapy instead of surgical invasive treatment, and achieved good therapeutic results.

## Case presentation

**Fig. 1:**
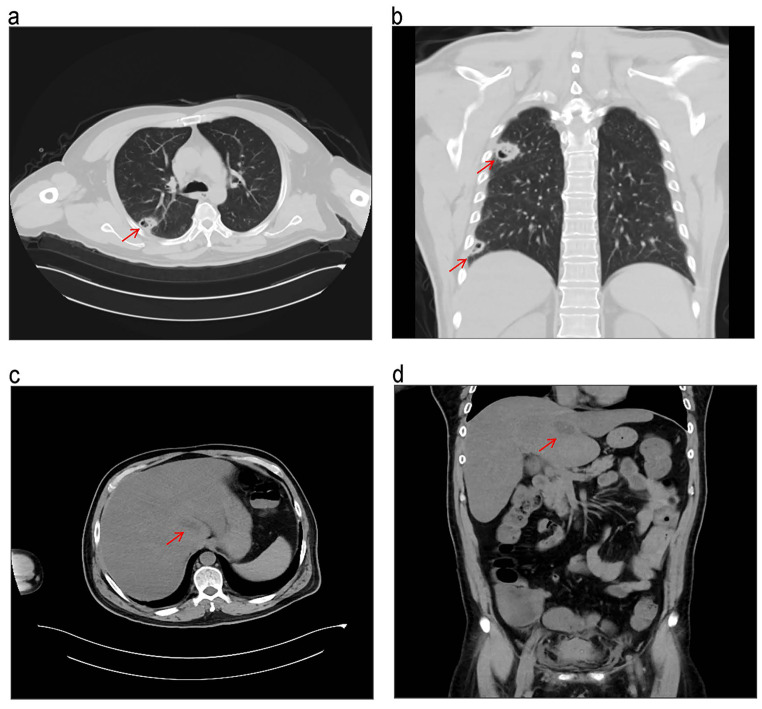
CT scans taken one day after admission demonstrating lung and liver findings. (**a**) and (**b**) Lung CT scans at admission show multiple nodules and patchy shadows across all lobes of both lungs. The most significant nodule, located in the lower lobe of the right lung, has a cross-section measuring about 3 cm × 2.2 cm, as indicated by the red arrow. (**c**) and (**d**) Hepatic CT scans reveal an increased volume of the caudate lobe and a low-density area(3.0 cm × 1.3 cm) in the caudate liver lobe, highlighted by the red arrow

A 53 years old man presented with intermittent high fever (39.6℃(135.04 °F), normal range,36 –37 °C (96.8 –98.6 °F)) and chills of 5 days. The patient did not exhibit gastrointestinal symptoms such as abdominal pain and diarrhea, nor did he have respiratory symptoms like cough and expectoration of sputum. There was no significant past medical history of liver disease or diabetes mellitus, intraperitoneal surgery, or immune deficiency. On admission, he had a temperature of 37.5 °C (99.5 °F), a blood pressure of 100/68(normal range, 60–90/90–140) mmHg, dyspneic (RR = 38 breaths/min, normal range, 16–20 breaths/min). Lung sounds were clear, and no rales, wheezing, or rhonchi were appreciated. The patient presented with tachycardia (heart rate, 154 beats per minute, normal range, 60–100 beats per minute) without murmur, rub, or gallop. Tenderness was elicited on the right upper quadrant. Murphy’s sign was not elicited. The liver and spleen were not palpable. Skin lesions were absent. arterial blood gas analysis showed a pH of 7.31(normal range, 7.35–7.45), PaO_2_ 118 (normal range, 80–100) mmHg under conditions of oxygen administration at 5 L/min via mask, PaCO_2_ 29.2 (normal range, 35–45) mmHg, cGlu 16.6 (normal range, 3.8–5.9) mmol/L, cLac 2.9(normal range, 0.5–1.6) mmol/L. A full blood count revealed WBC count was 17.77 (normal range, 3.5–9.5) ×10^9^/L with neutrophil predominance (90.1%). C reactive protein (CRP) and procalcitonin (PCT) were elevated to 234.35 (normal range, 0–5) mg/L and 57.62(normal range, 0–0.046) ng/mL, respectively. His platelet count was 18(normal range, 100–350) ×10^9^/L. Fibrinogen also was increased to 6.8 (normal range, 2–4) g/L. Laboratory results included a prothrombin time of 16.0 (normal range, 9.0–14.0) seconds, an INR of 1.45(normal range, 0.77–1.25), an APTT of 37.6(normal range, 24–35) seconds, and D-dimer of 17.54 (normal range, < 0.55) mg/L. Creatinine and blood urea nitrogen (URN) were elevated to 128.9(normal range, 57–97) umol/L and 9.8 (normal range, 3.1–8.0) umol/L, respectively. His Hba1c was 7.88% (normal range, 4.6 − 6.2%). HIV was negative. CT scan of the lung demonstrated that there were multiple nodules and patchy shadows in each lobe of both lungs, the largest of which was located in the lower lobe of the right lung, with a larger cross-section of about 3 cm  × 2.2 cm (Fig. 1). CT scan of the liver showed a low- density area(3.0 cm × 1.3 cm) in the caudate liver lobe (Fig. 1). Combined with the CT imaging, the patient was determined to have metastatic infection in the lungs, in addition to liver, the suspected source of infection. For a suspected liver abscess with ILAS, intravenous imipenem-cilastatin sodium (3 g/day) was started. Sepsis treatment was initiated. The patient had an increased oxygen requirement 4 h after admission. Under conditions of oxygen administration at 5 L/min via mask, arterial blood work done revealed an obviously decreased pH of 7.28 (normal range, 7.35–7.45), PaO_2_ of 63.5 (normal range, 80–100) mmHg, PaCO_2_ of 31.8 (normal range, 35–45) mmHg, actual bicarbonate HCO_3_^−^ of 15 (normal range, 22–30) mmol/L, cGlu of 18.6 (normal range, 3.8–5.9) mmol/L, cLac of 2.6 (normal range, 0.5–1.6) mmol/L. Initiation of continuous renal replacement therapy in patients with severe lactic acidosis.

**Fig. 2:**
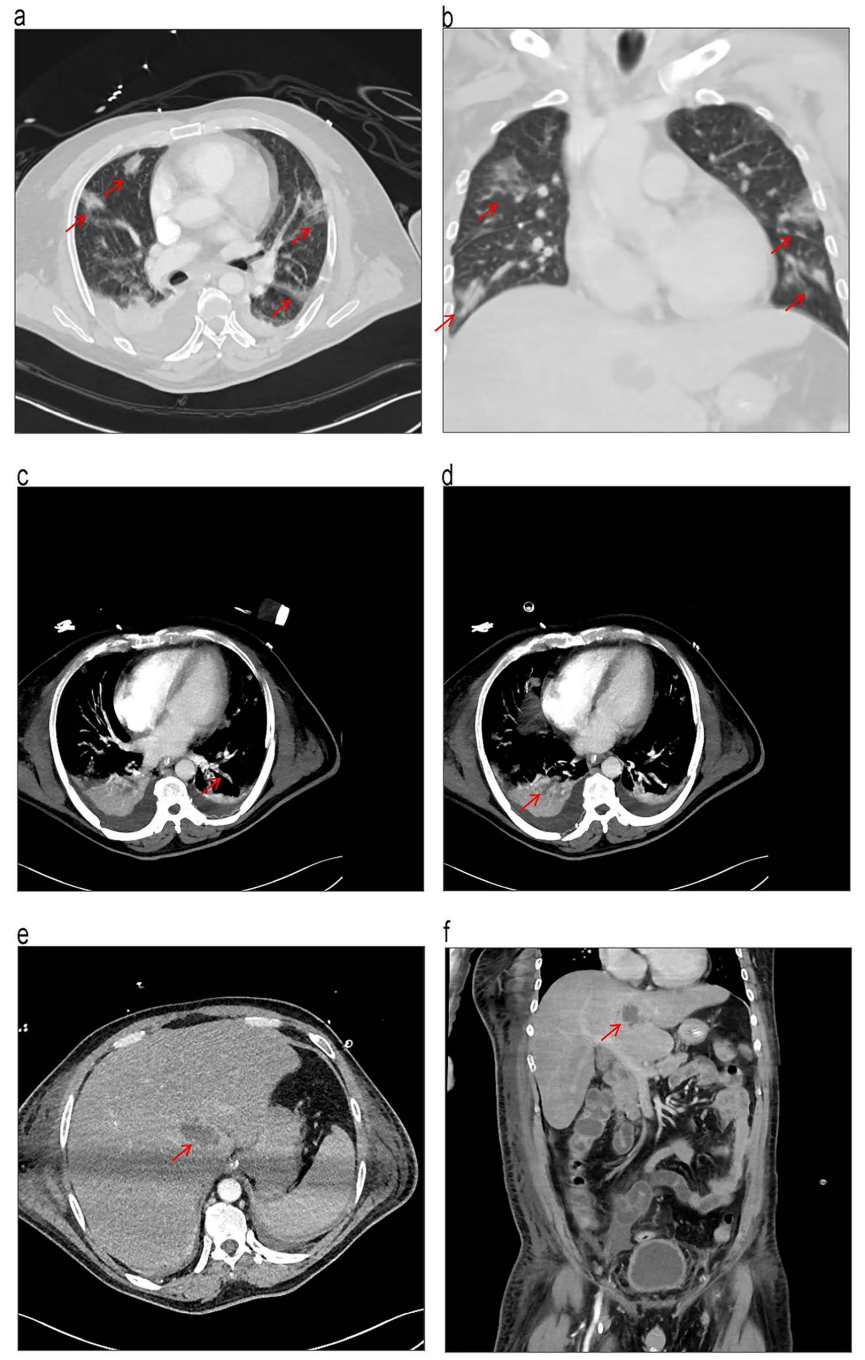
CT imaging findings eight days after admission. (**a**) and (**b**) Contrast-enhanced chest CTs reveal an increase in local infection, as indicated by the red arrow. (**c**) and (**d**) Improved CT scans suggest the presence of a pulmonary embolism. (**e**) and (**f**) Abdominal CT scans show an area of abnormal attenuation in the caudate lobe of the liver, measuring 3.3 cm × 2.1 cm × 2.2 cm

**Fig. 3:**
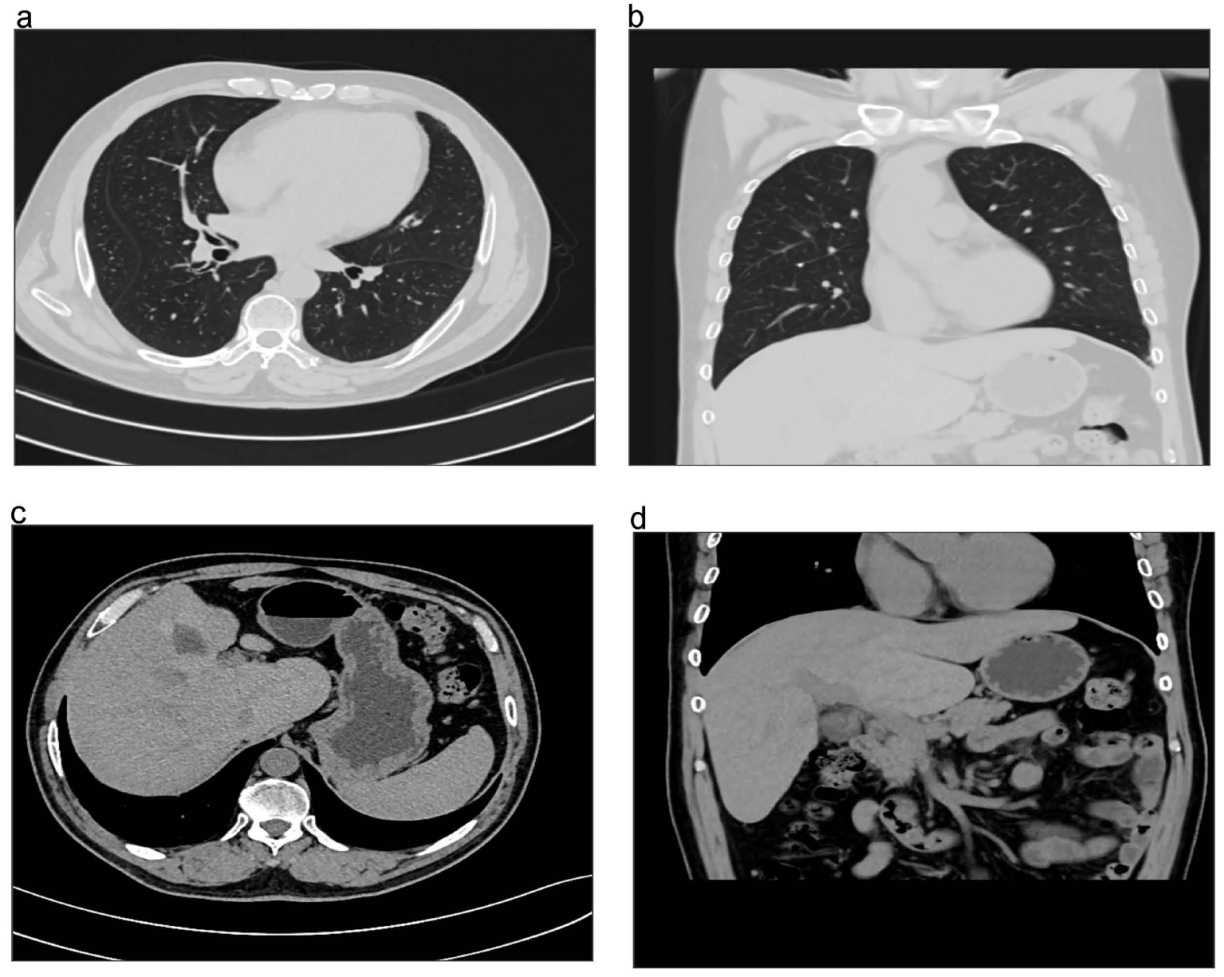
A repeat CT performed three months after discharge from the hospital showed resolution of lung and liver infections. (**a**-**d**) The CT images demonstrate the successful resolution of the infections in both the lung and liver, indicating a positive outcome of the treatment

He had a high fever of up to 39.9 °C (103.8 °F) 3 days after admission. Abdominal CT 3 days after admission revealed a 3.3 cm × 2.1 cm × 2.2 cm low-density area in the caudate lobe (Fig. S1). Chest CT demonstrated that increased local infection (Fig. S1). *K. pneumoniae* was isolated from blood culture, urine and phlegm, which was susceptible to imipenem, meropenem, ceftriaxone, amikacin, and ciprofloxacin. Eight days after admission, the improved CT examination suggested pulmonary embolism (Fig. 2), while cardiac ultrasound suggested pulmonary hypertension. Deep venous thrombosis was not observed by ultrasonography of the lower limbs. With the suspicion of liver abscesses, septic pulmonary embolism. The patient was treated with antibiotics and did not receive anticoagulation. Fifteen days after admission, there were no pulmonary embolism occurred (Fig. S2). The antibiotic therapy was successful (Fig. S3). The antibiotic regimen included intravenous Imipenem with cilastatin, levofloxacin, cefoperazone-sulbactam sodium and ceftazidime. The patient received antibiotics for 54 days and was hospitalized for 57 days. His data was collected through the follow in the outpatient department. A repeat CT performed 3 months after he was out of the hospital revealed resolution of the lung and liver infection (Fig. 3).

## Discussion

KPLA is a potentially life-threatening suppurating infection. The liver has double blood supplies including the hepatic artery and the portal vein. Since the gastrointestinal tract and the portal vein are linked, the pathogenic bacteria that cause liver abscesses primarily begin there. Diabetes mellitus and elderly male patients are most likely to develop abscesses [10, 11].

Our patient was a middle-aged man with elevated glycated hemoglobin, whose most obvious signs and symptoms at presentation (which included chills, rigors and fever) were suggestive of infection. The CT showed pulmonary abscess, pulmonary embolism and caudate liver abscess. Cultures of samples from blood, urine, and sputum were positive for *K. pneumoniae*. Our diagnosis was primarily based on the results from imaging and culture.

A review of the previously published cases of liver abscesses demonstrated that involvement of the right lobe was predominant (Table 1), rarely in the left lobe and bilateral lobes. However, the abscess in this case occurred in the caudate lobe. Rare infections bring great challenges to diagnosis and treatment. Liver abscesses contain both primary and secondary infections. In this case, the patient had no history of abdominal surgery or biliary tract infection, so we considered primary liver abscess. Hepatic abscess formation can occur through various routes, including biliary disorders, portal venous, hematogenous spread, direct infection and cryptogenic infection [12]. In this case, the absence of infection in the biliary system as seen on the liver CT scan does not support a biliary source of infection. Fig. S4 shows the main blood supply to the caudate lobe of the patient’s liver is derived from the portal vein, the blood supply of the portal vein to the caudate lobe is rich, and there is no obstruction in the inferior vena cava, excluding the possibility of retrograde infection. The likely reason for the infection in the caudate lobe of this patient may be due to *K. pneumoniae*, a conditional pathogen that entered through the portal vein, leading to a PLA. However, the patient had no evidence of intra-abdominal infection or intestinal infection, which does not align with the portal venous infection route. Therefore, we believe that the infection route for the caudate lobe liver abscess in this case is of cryptogenic origin. It was regrettable that the patient did not undergo endoscopy (gastroscopy, colonoscopy and small bowel endoscopy), serological tests or assessments of intestinal permeability to evaluate the integrity of the gastrointestinal mucosal barrier function.

**Table 1:** Summary of liver abscess sites from the last 20 years

Abscess location, *n* (%)	pathogenic bacterium	population	Country	Publication time	Publisher
Right lobe 54 (69.2); Left lobe 11(14.1); Both lobe 4(5.1); not documented 9(11.5)	Not Applicable	78	USA	2004	Rahimian J, et al. [13].
Right lobe 64 (58.2); Left lobe 33(30); Both lobe 13(11.8)	*K.* pneumoniae	110	Taiwan	2008	Lee SS, et al. [5].
Right lobe 157 (71); Left lobe 42(19); Edge between left and right lobes2(1); Both lobe 21(9)	*K.* pneumoniae	222	China	2013	Shen D, et al. [14].
Right lobe 35 (77.7); Left lobe 7(15.6); Both lobe 3(6.7)	*K.* pneumoniae	45	China	2015	Qu TT, et al. [15].
Right lobe 22 (70.9)	*K.* pneumoniae	31	France	2018	Rossi B, et al. [12].
right lobe	*K.* pneumoniae	1	Sri Lanka	2018	Premathilake PNS, et al. [16]
Right lobe 95 (81.2); Left lobe 22 (18.8)	*K.* pneumoniae	117	China	2019	Zhang S, et al. [17].
Right lobe 43(82.7); Left lobe 7(13.5); Both lobe 2(3.9)	Not Applicable	52	China	2020	He S, et al. [18].
Right lobe 1050 (72.7); Left lobe 236 (16.3); Both lobe 158(10.9)	Not Applicable	1444	China	2020	Yin D, et al. [4].
Right lobe 75 (75); Left lobe 16(16); Both lobe 9(9)	*K.* pneumoniae	100	China	2020	Ren Y, et al. [19]
multiple liver lesions	*K.* pneumoniae	1	USA	2021	Bradley ME, et al. [20].
Right lobe 90 (66.7); Left lobe 34 (25.2); Both lobe 11(8.1)	*K.* pneumoniae	135	China	2021	Li S, et al. [7].
Right lobe 162 (54.7); Left lobe 63 (21.3%); Both lobe 71(24.0)	Not Applicable	296	Spain	2023	Justo I, et al. [21].

Persistent thrombocytopenia is related to poor prognosis in Intensive Care Unit (ICU) patients [22]. The patient’s platelet count is very low, amounting to 6 × 10^9^/L. *K. pneumoniae* infections can create thrombocytopenia. Platelets accelerate the development of hyperinflammation and microthrombosis. Patients with diabetes and ILAS showed elevated platelet reactivity. The main factor in the development of a widespread infection is the formation of a circulating platelet-leukocyte complex, which is facilitated by platelets [23]. Lancet Infectious Diseases reported that *K. pneumoniae* liver abscess is as a new invasive syndrome. The main risk factors for the development of metastatic infection in *K. pneumoniae* liver abscess are diabetes, the high virulence of the isolated strains (serotypes K1 and K2), and the expression of the gene r*mpA* [6]. The classification of the invasive liver abscess syndrome included endophthalmitis, empyema, septic pulmonary embolism, meningitis, osteomyelitis, psoas muscle abscess, chest wall abscess, and necrotizing fasciitis [6, 24]. In this case, he developed a septic pulmonary embolism.

Standard treatment for patients with liver abscesses is using adequate antibiotics (third-generation cephalosporins, fluoroquinolones, carbapenems) and drainage [25]. Carbapenem antibiotics should be preferred when the pathogen is unclear, sepsis, or septic shock. The caudate lobe of the liver has the dual characteristics of deep anatomical position and small size. Drainage treatment of caudate lobe liver abscess is very easy to cause damage to the surrounding structures and may lead to greater morbidity or even mortality. Therefore, some scholars suggest conservative treatment [26]. Currently, there are no guidelines for the drainage of caudate lobe liver abscesses. A review of past cases regarding the drainage treatment of caudate lobe abscesses found that abscesses with a maximum cross-sectional area greater than 9.86 cm² were treated with drainage (table S1). The maximum cross-sectional area of the abscess in this patient was 5.7 cm². Therefore, this patient was treated with antibiotic therapy for infection and was successfully cured.

## Conclusion

The rarity of cases of *K*. pneumoniae caudate lobe abscess has also contributed to the lack of formal guidance. We describe the case of a *K. pneumoniae* caudate liver abscess with ILAS who was successfully treated with antibiotics with CRRT. Moving forward, we suggest that the patients with cryptogenic *K. pneumoniae* liver abscess are advised to undergo examination of intestinal barrier function (gastroscopy, small bowel endoscopy and tests for intestinal permeability). The study indicates that in patients with *K. pneumoniae* liver abscess, a caudate liver abscess size of ≤ 9.86 cm² may be characteristic of those suitable for conservative treatment of invasive liver abscess syndrome.

### Electronic supplementary material

Below is the link to the electronic supplementary material.

Supplementary Material 1

## Data Availability

Upon reasonable request, the corresponding author will share all the data.

## Abbreviations

CRP: C reactive protein
CRRT: Continuous renal replacement therapy
CT: Computed tomographic
ICU: Intensive Care Unit
ILAS: Invasive liver abscess syndrome
KPLA: Klebsiella pneumoniae-caused liver abscess
PCT: Procalcitonin
PLA: Pyogenic liver abscess
URN: Urea nitrogen
