# Supplementary material for: Liver abscess in the caudate lobe caused by Klebsiella pneumoniae: a rare case report and literature review
Source: BMC Infect Dis. 2024 Jul 19;24:708. doi: 10.1186/s12879-024-09569-6 (PMC11264778; doi:10.1186/s12879-024-09569-6)
Supplement: Supplementary file 1 — Supplementary Material 1 [file 12879_2024_9569_MOESM1_ESM.docx]

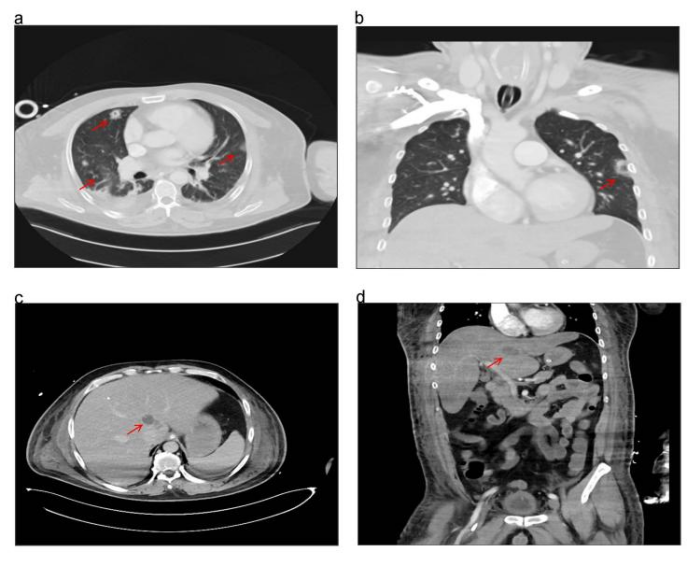


**Fig. S1. CT imaging findings three days after antibiotic treatment.** **(a)** and **(b)** Contrast-enhanced chest CTs show increased local infection, as indicated by the red arrow. **(c)** and **(d)** Abdominal CT scans, taken three days after admission, reveal a low-density area measuring 3.3 cm × 2.1 cm × 2.2 cm in the caudate lobe, highlighted by the red arrow.


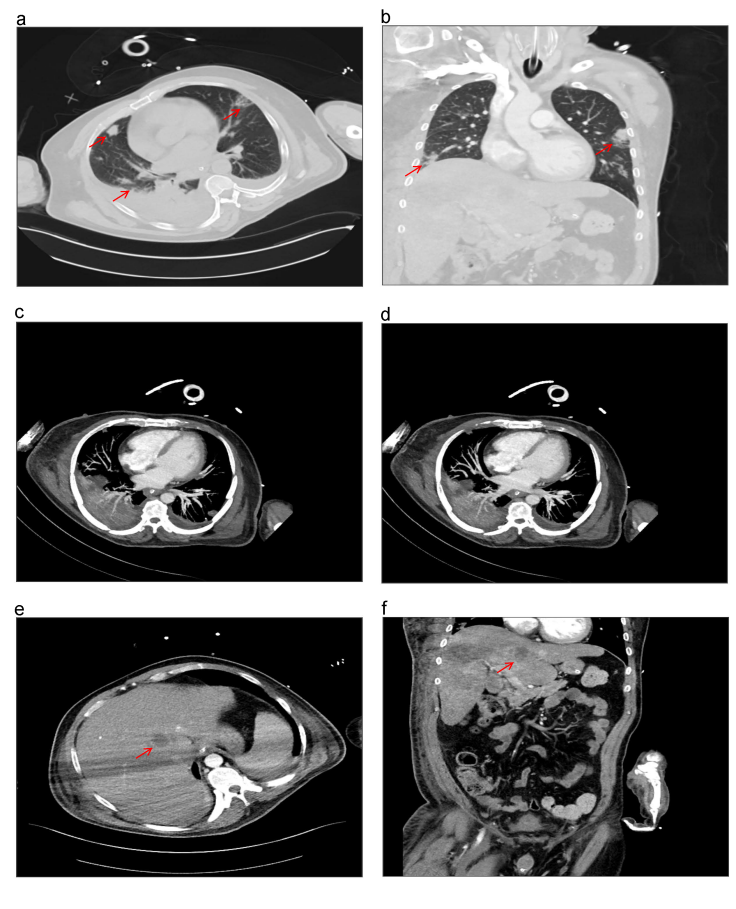


**Fig. S2. CT imaging findings 15 days after starting antibiotics. (a)** and **(b)** Chest CTs demonstrate the resolution of the lung infection, indicated by the red arrow. **(c)** and **(d)** Enhanced CTA scans show no evidence of pulmonary embolism. **(e)** and **(f)** Abdominal CT scans reveal that the infection focus is smaller than previously observed, highlighted by the red arrow.


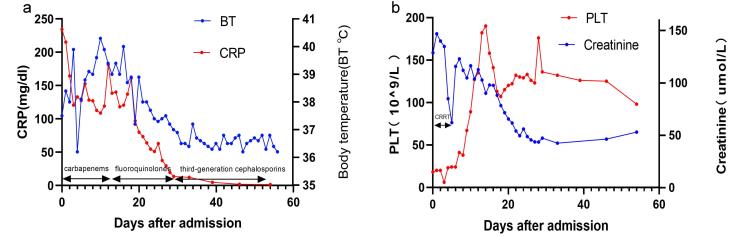


**Fig. S3. Clinical course after admission with key laboratory value trends. (a)** Illustrates the changes in platelet count throughout the treatment period. **(b)** Monitors the levels of creatinine and C-reactive protein (CRP) following admission. This figure highlights the patient's response to treatment via crucial blood test indicators.


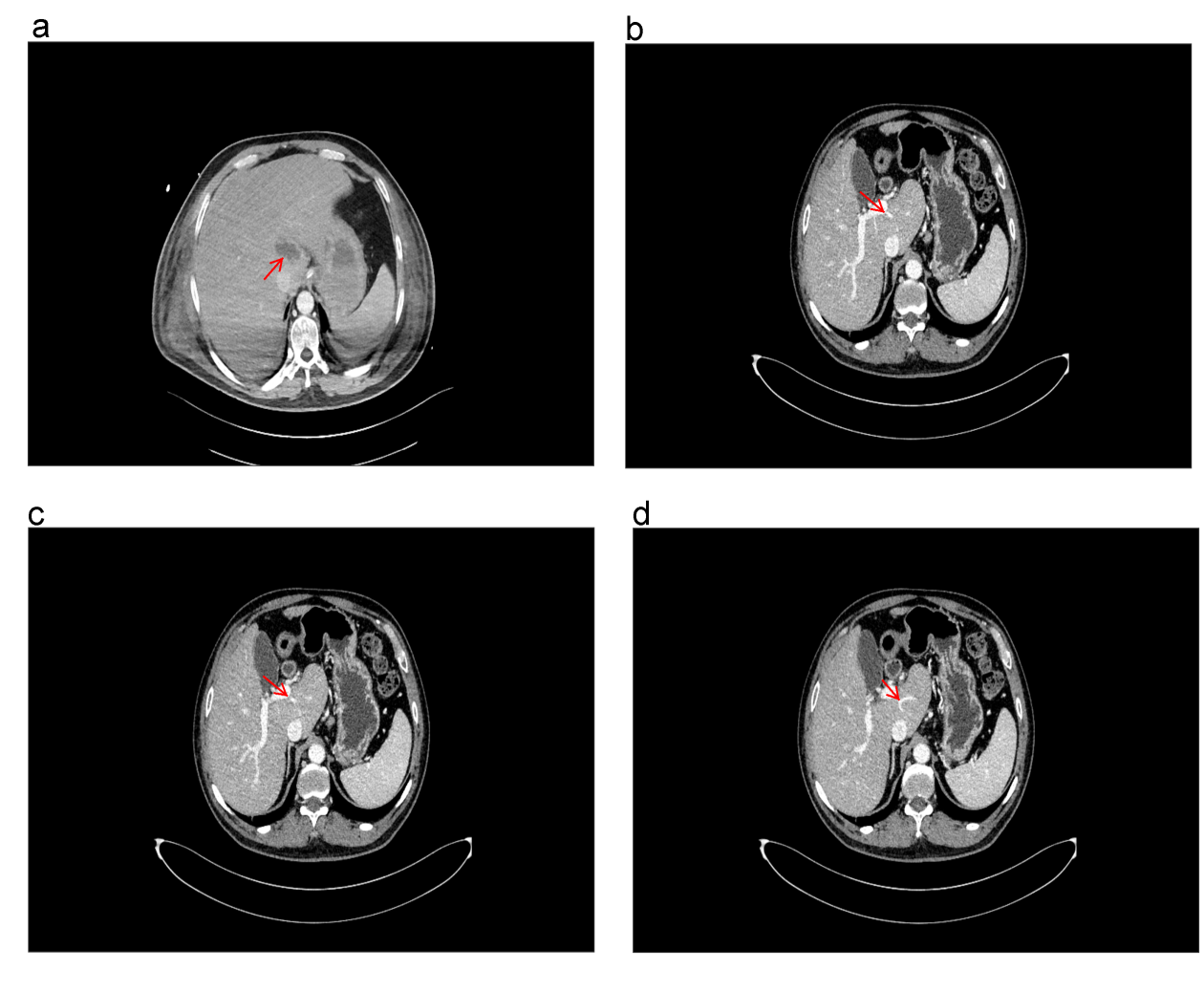


**Fig. S4. Visualization of the caudate lobe's blood supply and abscess location via hepatic CT scan. (a)** Reveals a low-density area within the caudate lobe, marked by the red arrow. **(b), (c),** and **(d)** Show the distribution of portal vein branches throughout the caudate lobe, also indicated by the red arrow. The abscess's location within the caudate lobe aligns with regions densely supplied by portal vein branches.

Table S1 Summary of caudal lobe liver abscess puncture drainage abscess size

| site of action | the size of the lesion | area | pathogenic bacterium | Publication time | Publisher |
| --- | --- | --- | --- | --- | --- |
| caudate lobe | 3 cm ×4.2 cm | 9.86cm^2^ | E. coli, K. pneumoniae | 2013 | Al Amer NA. et al [1] |
| caudate lobe | 5.9cm× 6.3cm | 29.62cm^2^ | Pus culture revealed no growth | 2021 | Neetha V. et al [2] |
| caudate lobe | 4.1cm× 3.8cm | 12.05 cm^2^ | Streptococcus intermedius | 2022 | Xia F. .et al [3] |
| caudate lobe | 6.0cm× 6.0cm | 28.27 cm^2^ | Pus culture revealed no growth | 2022 | Zanwar S. et al [4] |
| caudate lobe | 4.5cm ×5.1 cm | 18.57 cm^2^ | K. pneumoniae | 2023 | Kaneko J. et al [5] |

1. Al Amer NA, Abd El Maksoud WM: Abscess of the caudate lobe of the liver, a rare disease with a challenging management: a case report. *J Biomed Res* 2013, 27(5):430-434.

2. V N, M RB, S V: **A rare case of caudate lobe liver abscess**. *International Surgery Journal* 2021, **8**(9).

3. Xia F, Zhu P, Chen XP, Zhang BX, Zhang MY: Liver abscess in the caudate lobe caused by a fishbone and treated by laparoscopy: a case report. *BMC Surg* 2022, 22(1):6.

4. Zanwar S, Agrawal A: Endosonography-Guided Caudate Lobe Liver Abscess Drainage: A Case and Review of Methods. *Journal of Digestive Endoscopy* 2022, 13(02):119-124.

5. Kaneko J, Tamakoshi H, Watahiki M, Kusama D, Niwa T, Takinami M, Yamada T: **Endoscopic ultrasound-guided caudate lobe liver abscess drainage**. *Endoscopy* 2023, **55**(S 01):E1099-E1100.
